# Microneedle-Facilitated Intradermal Proretinal Nanoparticle Delivery

**DOI:** 10.3390/nano10020368

**Published:** 2020-02-20

**Authors:** Benchaphorn Limcharoen, Pattrawadee Toprangkobsin, Marius Kröger, Maxim E. Darvin, Titiporn Sansureerungsikul, Teeranut Rutwaree, Supason Wanichwecharungruang, Wijit Banlunara, Jürgen Lademann, Alexa Patzelt

**Affiliations:** 1 Department of Pathology, Faculty of Veterinary Science, Chulalongkorn University, Pathumwan, Bangkok 10330, Thailand; ben.limcharoen@gmail.com (B.L.); Wijit.k@chula.ac.th (W.B.); 2 Department of Dermatology, Venereology and Allergology, Center of Experimental and Applied Cutaneous Physiology (CCP), Charité—Universitätsmedizin Berlin, Corporate Member of Freie Universität Berlin, Humboldt-Universität zu Berlin, and Berlin Institute of Health, 10117 Berlin, Germany; marius.kroeger@charite.de (M.K.); maxim.darvin@charite.de (M.E.D.); juergen.lademann@charite.de (J.L.); 3 Nanotec-CU Center of Excellence on Food and Agriculture, Department of Chemistry, Faculty of Science, Chulalongkorn University, Pathumwan, Bangkok 10330, Thailand; t_pattrawadee@hotmail.com (P.T.); supason.p@chula.ac.th (S.W.); 4 Mineed Technology, 201-201 Tower C Thailand Science Park, Pathum Thani 12120, Thailand; san.titiporn.chulalongkorn@gmail.com (T.S.); teeranut.rut@gmail.com (T.R.); 5 Center of Excellence in Advanced Materials and Biointerfaces, Chulalongkorn University, Bangkok 10330, Thailand

**Keywords:** microneedle, retinal, nanocarrier, dermis, drug delivery

## Abstract

Topical retinoid treatments stimulate biological activities in the skin. The main physical barrier, which limits the efficacy of transdermal drug delivery, is the stratum corneum. Proretinal nanoparticles (PRN) have already been proven to efficiently deliver retinal into the epidermis. In the present study, two transdermal drug delivery systems, microneedles (MN) and PRN, were combined to directly target the dermis. The microchannels induced by the MN, the PRN localization in the microchannels and the skin closure kinetics were investigated by non-invasive imaging techniques, such as dermoscopy, optical coherence tomography and multiphoton tomography. Additionally, the amount of retinal in the epidermis and dermis after application in three different forms (PRN-Loaded microneedles, PRN suspension or conventional retinal solution) was compared. All imaging techniques confirmed the formation of microchannels in the skin, which were partly still detectable after 24 h. Multiphoton tomography showed the release of PRN from the MN within the microchannels. The recovered retinal concentration in the dermis was significantly higher when applied via PRN-loaded microneedles. We hypothesized that this platform of PRN-loaded microneedles can provide a rapid and efficient administration of retinal in the dermis and could be of benefit in some skin conditions such as atrophic scar or photo-aged skin.

## 1. Introduction

Retinoids are frequently topically applied to improve several skin conditions. They are known to have an influence on dermal collagen synthesis [1], are utilized to reduce signs of skin aging [2,3,4] and have been shown to provide smooth atrophic acne scars [5,6]. Moreover, retinoids are known to activate fibroblasts, to increase type I procollagen and to decrease matrix metalloproteinase (MMP) expression in skin damaged by ultraviolet (UV) irradiation [1,7], to improve the skin texture and to reduce skin discoloration [8]. Retinal (retinaldehyde; RAL), a natural precursor of retinoic acid, has been shown to exert the biological activities of retinoids and to be likewise beneficial for the treatment of photoaging [9] and acne scarring [10]. Although application of retinal is useful in clinical dermatology and cosmetology, there are still some limitations concerning the topical application such as its photochemical instability and irritation in long-term application. Moreover, the low skin bioavailability of retinoids represents a problem. All-*trans*-retinoic acid, e.g., is accumulated mainly in the epidermis and provides only relatively low concentrations in the dermis [11]. The skin bioavailability of the commercially available all-*trans*-retinoic acid is only 5% [12]. The main biological barrier for drug delivery is the stratum corneum, which is formed by corneocytes embedded in the structural lipid matrix with a thickness of 10–20 µm [13,14], and which predominantly limits the efficacy of transdermal drug delivery systems [15]. New microneedle technologies have been suggested to tackle the limitations of customary transdermal drug delivery systems. Microneedles (MN) consist of an array of micron-sized needles with a length of up to 1000 µm [16] to overcome the stratum corneum and to facilitate the active substances to enter the viable epidermis or dermis. The MN method is a minimally invasive procedure. Different biodegradable and water-soluble polymers such as sodium hyaluronate [17,18], polyvinylpyrrolidone (PVP) [19,20] and polyvinyl alcohol (PVA) [21] have been used to fabricate dissolvable MN. Recently, proretinal nanoparticles (PRN) have been introduced. They showed a promising capability to overcome the instability of retinal and they were able to release retinal in the skin at skin pH [22]. However, nanoparticulate systems have been proved to efficiently deliver retinal mainly into the epidermis, which is the upper part of the skin [22] or the hair follicles [23]. Topical drug delivery of retinal into the dermis represents a further challenge, which potentially can be managed by the application of PRN-loaded microneedles. It is hypothesized that PRN in the dissolving MN can slowly release retinal into the dermis. Therefore, for the present study, dissolvable PRN-loaded MN have been developed to deliver PRN directly into the dermis. The aim of the investigation was to visualize both the formation of microchannels induced by the PRN-loaded MN and the resealing of the skin by non-invasive techniques including dermoscopy, optical coherence tomography (OCT) and multiphoton tomography (MPT) as to demonstrate the possibility of the combination of two delivery systems to deliver retinaldehyde (retinal; RAL) into the dermis. Moreover, the retinal concentration in both the epidermis and the dermis was compared after topical application of retinal in three different forms (PRN-loaded MN, PRN, and conventional RAL). All experiments were performed on ex vivo porcine skin which is a suitable skin model [24].

## 2. Materials and Methods

### 2.1. Fabrication of the Dissolving PRN-Loaded Microneedles

PRN was prepared as previously described with some minor adjustments [19]. Briefly, chitosan (CS, molecular weight of ~40,000–50,000 Da, Taming Enterprise, Samut Sakhon, Thailand) was dissolved in 0.05% acetic acid, and the pH of the obtained solution was adjusted to 5.9 using NaOH. The final solution contained 45 mg CS in 19.0 mL solution. Then, cold retinal (15 mg, Sigma Aldrich, St. Louis, MO, USA, in 1.0 mL of ethanol) was slowly added dropwise to the cold CS suspension (5 °C) under light-proof condition while the mixture was continuously ultrasonicated (40 kHz) under nitrogen atmosphere. The obtained PRN suspension was then freeze-dried. The obtained dry PRN (60 mg) was mixed into the 2 mL polymer solution (4% sodium hyal, uronate (injection grade, Shandong Focuschem Biotech Co., Ltd., Shandong Sheng, China), 4% polyvinylpyrrolidone (Sigma-Aldrich,) and 2% maltose (Sigma-Aldrich). MN patch was fabricated using the obtained mixture, according to the previously described platform under the particle-free atmosphere (Clean room class 1000) [25]. In brief, the mixture was poured into the mold and left under moisture control atmosphere of 5% humidity until the intact hydrogel was formed, then the water penetrable cellulose membrane was attached. Subsequently, the mold was removed and an array of needle-shaped hydrogel sitting on the cellulose membrane was dried in a light-proof, low pressure and moisture-controlled (≤2%) chamber to obtain PRN-loaded microneedles with a cellulose base sheet. The PRN-loaded MN patch obtained was a 5 × 5 mm patch containing array of 10 × 10 needles of tetragonal pyramidal shape with 200 × 200 µm base and 650 µm needle height. The amount of retinal loaded in the 10 × 10 needles part of each MN patch was quantified by dissolving the obtained PRN-loaded MN in acidic water under N_2_ atmosphere. Then retinal was extracted from the solution (3 times) with ethyl acetate under saturated N_2_ to prevent retinal degradation. The amount of retinal in the obtained ethyl acetate extract was quantified by UV absorption spectroscopy using λ_max_ of 330 nm. A calibration curve was constructed using standard retinal solutions prepared in ethyl acetate. A stereomicroscope (Olympus DP22, Tokyo, Japan) was used to observe the morphologies and dimensions of the MN.

### 2.2. Experimental Design of Topical Applications

Fresh ears from 6-months-old German domestic pigs without any skin lesions were obtained from a local abattoir. The used protocol was approved by the Veterinary Board of Control, Dahme-Spreewald. The porcine ears were cleaned under running tap water and dried with paper towels. Hairs were trimmed to a length of 1 mm. Four areas of 1.5 × 3 cm^2^ were defined. One skin area remained untreated as control, the other skin areas were treated either with PRN-loaded MN, PRN suspension (at 3.33 mM of retinoid in water) or the freshly prepared 0.1% *w/v* conventional retinal solution in ethanol (equivalent to 3.33 mM retinoid; conv. RAL).

The test substances were applied to the skin areas, which were demarcated by using a silicon barrier (Marabu Window Color, Marabu GmbH, Bietigheim-Bissingen, Germany) to prevent the lateral spreading of the applied substances. The 20 µL/cm^2^ of PRN or conv. RAL suspension were topically applied (equivalent to 12 µg of retinal/4.5 cm^2^) to two different skin areas and were distributed homogeneously with 2 min of 50 Hz massage appliance (Novafon Pro soundwave appliance, Weinstadt, Germany). Then, the skin samples were incubated for 4 h at room temperature. PRN-loaded MN patches were manually pressed into the third test area. Then the patch was held in place for 5 min with some gentle massage motions and gentle pressure using the finger tip, then the base of the patch was peeled off. Six patches of MN were used on an area of 4.5 cm^2^. The peeled off base was subjected to microscopic examination to make sure that all needles have been detached and left in the skin. Each experiment was performed on six independent pig ears (n = 6). Due to the light sensitivity of retinoids, light exposure was avoided in all experiments.

### 2.3. Optical Methods to Study Microchannel Formation and Kinetics and PRN Release after the Application of PRN-Loaded MN

#### 2.3.1. Dermoscopy

To investigate the ex vivo insertion ability of PRN-loaded MN, dermoscopic examination was performed immediately (0 h), 4 h and 24 h after MN administration. A computerized polarized light videodermatoscope (FotoFinder Dermoscopy equipped with Medicam 800 HD; FotoFinder Software version 119.612.01.2011 LK/SM, Bad Birnbach, Germany) with magnification factors of ×20 to ×70 int ×10 increments lens was utilized to investigate at least three test areas of skin treated with PRN-loaded MN for each time point. As polarized light was used, no preparation of the area under examination was necessary.

#### 2.3.2. Optical Coherence Tomography (OCT)

OCT was used to visualize the morphologic changes of the superficial skin layers after MN administration and to confirm the formation of microchannels following the application of MN on porcine skin. At least three test areas of skin treated with PRN-loaded MN for each time point were investigated. The test areas of MN insertion were scanned with OCT (Vivosight OCT Scanner, Michelson Diagnosis Ltd., Kent, UK) after dermoscopy over an area of 6 × 6 mm, at an imaging depth of 1 mm and an optical resolution of <7.5 µm laterally and <5 µm axially immediately after MN administration, and after 4 h and 24 h. No preparation of the skin surface was required for OCT scanning. The function ‘multi-1’ setting automatically generated 60 lateral scans of 6-mm length every 100 µm of lateral scanning of the axial OCT scans resulting in two-dimensional cross-sectional images and en-face views. The OCT images were assessed by naked eye for morphological changes of the epidermis and the dermis immediately, 4 h and 24 h after single insertion.

#### 2.3.3. Multiphoton Tomography (MPT) with Fluorescence Lifetime Imaging Microscopy (FLIM)

In order to verify the position of PRN dissolved from MN, images of the epidermis and dermis were acquired by means of multiphoton tomography (MPT) (Dermainspect, JenLab GmbH, Jena, Germany) utilizing a tunable (710–920 nm) femtosecond titanium sapphire laser (Mai Tai XF, Spectra Physics, Santa Clara, CA, USA). Full thickness skin samples of 1 × 1 cm^2^ in size were prepared for the experiment after PRN-loaded MN insertion. At least three test areas of skin treated with PRN-loaded MN for each time point were investigated.

To detect autofluorescence, the excitation wavelength used for this study was at 760 nm. The laser generated 100-fs pulses at a repetition rate of 80 MHz. Due to two-photon absorption, a 410–680 nm bandpass filter was utilized for the autofluorescence detection. The microchannels created by the PRN-loaded MN were studied using fluorescence lifetime imaging microscopy (FLIM) for the detection of changes in fluorescence lifetime of intrinsic autofluorescent compounds. Thereby, FLIM data were analyzed by the SPCImage software (version 4.2, Becker & Hickl, Berlin, Germany) incorporated in the Dermainspect system. Fluorescence decay in each pixel was fitted with a sum of two exponentials (fast and slow) using the weighted least squares method with a fixed shift value [26], the intensity threshold was chosen depending on the image quality, and binning was set to 3, analyzing the pixel of interest and 48 neighboring pixels to minimize artifacts. The obtained lifetime (*τ*_1_ and *τ*_2_) and amplitude (*a*_1_ and *a*_2_) values were further exported and used for the evaluation of lifetime distributions and image segmentation. The average lifetime was defined as *τ*_m_ = (*a*_1_*τ*_1_ + *a*_2_*τ*_2_)/(*a*_1_ + *a*_2_). The scanning modality of MPT-FLIM images was x-y scanning, resulting in z-stacks of horizontal images from the stratum corneum to the dermis. The z-stacks were taken by moving the objective in the z-direction, thus scanning at different depths in the skin at 10 µm increments. The utilized TPT-FLIM device was described elsewhere [20,27].

### 2.4. Skin Penetration of Retinal from PRN-Loaded MN, PRN and Conv. RAL

Skin penetration of retinal was investigated after topical application of PRN-loaded MN, PRN and conventional RAL on ex vivo porcine ear skin, as described below.

#### 2.4.1. Extraction of Epidermis and Dermis

After topical application of PRN-loaded MN, PRN and conventional RAL to the different test areas, epidermis and dermis were extracted to quantify the retinal concentration in both compartments. To separate epidermis from dermis, full-thickness skin was dissected from the underlying cartilage by using a scalpel and heated for 1 min on a stainless-steel heating plate at 60 °C [28]. Subsequently, epidermis could be entirely peeled from dermis with forceps. Epidermis and dermis from each treatment group were homogenized using a TissueLyser II (Qiagen, Venlo, The Netherlands) for 1 min at 30 Hz and placed into sterile 2 mL round-bottom tubes containing a 0.5 cm diameter stainless steel bead. Subsequently, all samples were continuously disrupted another 1 min with 1 mL of ethanol at pH 3 (Ethanol UVASOL, Merck, Darmstadt, Germany). Each sample was placed in a single test tube, which was filled with 2.14 mL ethanol at pH 3. Then, all tubes were ultrasonicated for 10 min (Sonorex Super RK102H, Bandelin Electronic, Berlin, Germany) and centrifugated at 4000 rpm for 10 min at 20 °C (Hettich^®^ Universal 320/320R centrifuge, Sigma Aldrich). Retinal concentration in the epidermis and dermis from all treatment groups was determined by UV-Vis spectroscopy.

#### 2.4.2. UV-VIS Spectroscopy for Quantification of Retinal in the Skin

After extraction, the absorption spectra of retinal in the supernatant were recorded with a Lambda 650 S UV-visible spectrometer (Perkin Elmer, Uberlingen, Germany) at 25 °C in the range of 250–500 nm using a quartz cuvette with 10 mm path length (Quartz Suprasil, Hellma Analytics, Müllheim, Germany). The band maximum of the retinal is at 380–400 nm. The results obtained from the skin probes from PRN-loaded MN, PRN and conv. RAL were compared with the untreated skin areas, which served as control. The amount of recovered retinal in epidermis and dermis from all formulations was calculated using the standard reference curve prepared of retinal standards.

### 2.5. Statistical Analysis

To analyze the data, the one-way ANOVA test followed by the Tukey’s multiple comparisons test was utilized to investigate the differences of recovered retinal concentration in epidermis and dermis between groups using GraphPad software (Graphpad Prism 7, GraphPad Software, San Diego, CA, USA). Differences were considered significant at *p*-value < 0.05.

## 3. Results

### 3.1. Morphology of PRN-Loaded MN

**Figure 1:**
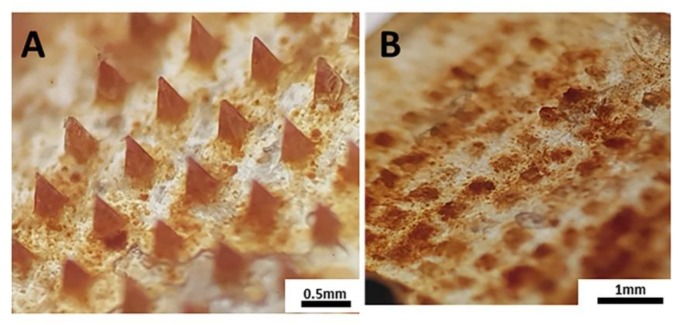
Characterization of PRN-loaded MN applied to ex vivo porcine ear skin. Optical image of (**A**) PRN-loaded MN patch before application and (**B**) PRN-loaded MN patch after application.

PRN were successfully prepared as the reddish orange colored particles with the size distribution of approximately 240 ± 29 nm obtained from the DLS analysis. SEM image (Figure S1) reveals spherical particles with the dry size of around 1 µm. The obtained PRN were used for the preparation of the PRN-loaded MN patches. After fabrication, microscopy confirmed that the MNs array had a dimension of 0.5 × 0.5 cm with 10 × 10 needles in each patch. Each needle had a tetragonal pyramidal shape with a sharp-pointed tip. The dimension of each tetragonal pyramidal needle was 200 × 200 µm at the base, the height of each needle was 650 µm (Figure 1A). The yellow-orange PRN [22] were highly concentrated at the needles, forming a yellowish orange tip on each MN (Figure 1A). The amount of retinal loaded in the 10 × 10 needles part of each MN patch was 2.08 ± 0.55 µg. After application, the complete array of MN became detached from the base and remained in the skin (Figure 1B).

### 3.2. Optical Methods to Study Microchannel Formation and Kinetics and PRN Release after the Application of PRN-Loaded MN

#### 3.2.1. Dermoscopy

**Figure 2:**
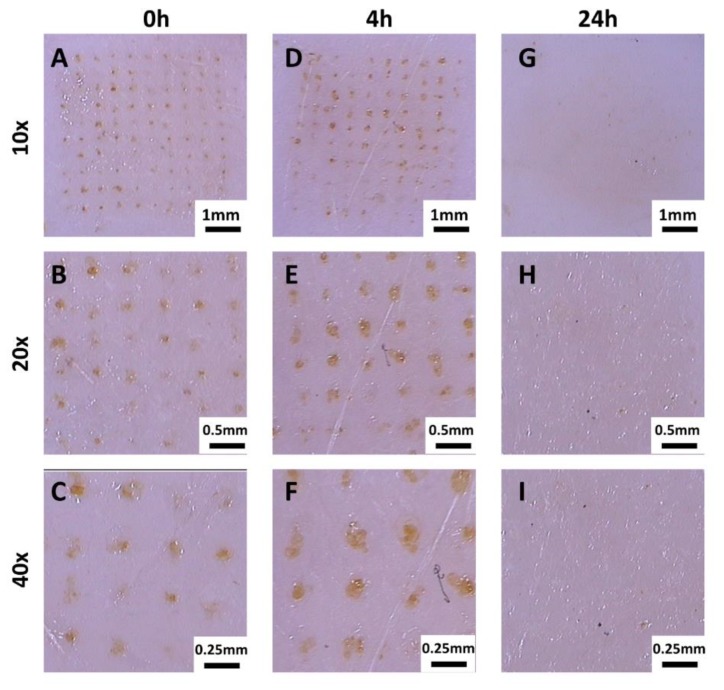
Dermoscopic images of porcine skin treated with PRN-loaded MN measured at different magnifications. (**A**–**C**) Dermoscopic images obtained immediately after topical application of PRN-loaded MN. (**D**–**F**) Dermoscopic images obtained 4 h after topical application of PRN-Loaded MN. (**G**–**I**) Dermoscopic images obtained 24 h after topical application of PRN-loaded MN.

Dermoscopic examination of the test areas was performed immediately, 4 h and 24 h after topical application of PRN-loaded MN. Corresponding dermoscopic images of the porcine skin taken immediately, 4 h and 24 h after application are shown in Figure 2. The yellow dots, which are visible in the skin after treatment with PRN-loaded MN, represent the tips of the MNs. They are only visible in the skin samples, which were measured immediately and 4 h after topical application (Figure 2A–F). At 24 h after topical application, the yellow dots were not visible anymore (Figure 2G–H).

#### 3.2.2. Optical Coherence Tomography (OCT)

**Figure 3:**
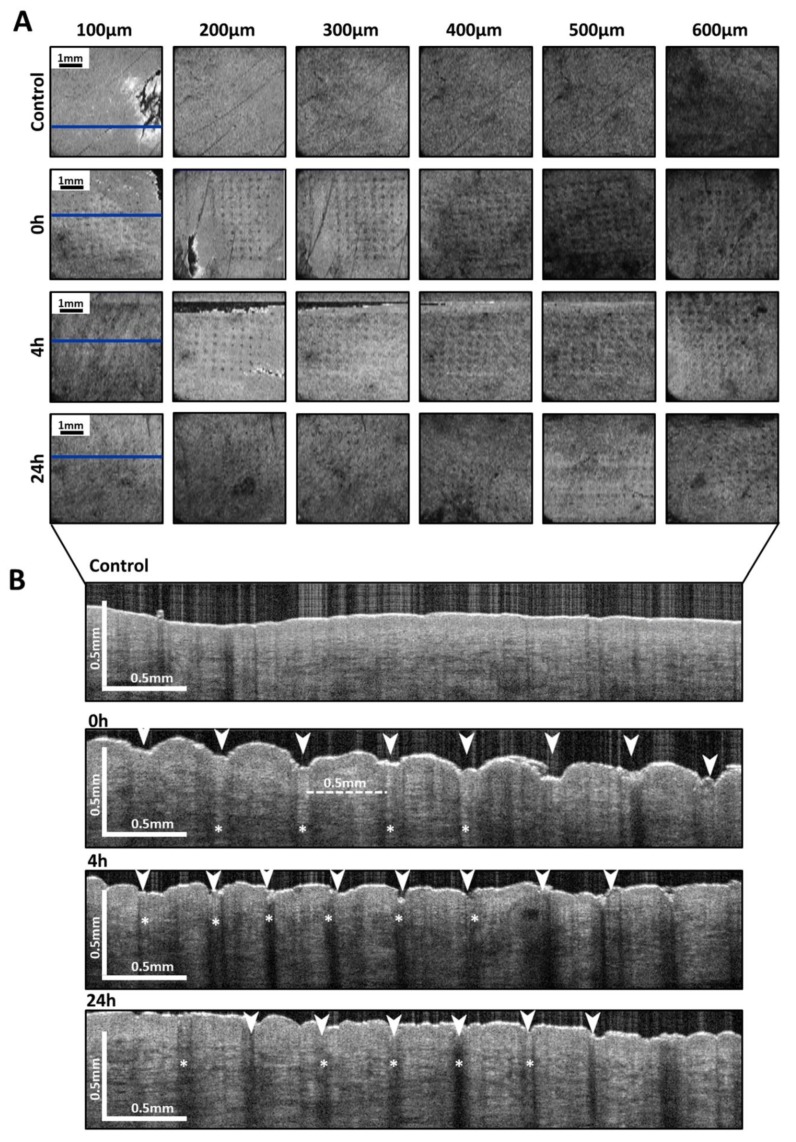
Optical coherence tomography images immediately, 4 h and 24 h after insertion of PRN-loaded MN compared with the untreated skin (control). (**A**) En-face OCT images (XY plane) in projection view of the skin surface obtained from different depth ranges from 100–600 µm beneath the skin surface. Scale bar represents 1 mm. The blue line represents one row of the microneedle array, which was subjected to be shown in the cross-sectional view. (**B**) Cross-sectional OCT images (XZ plane) showing the ability of MN to penetrate in ex vivo porcine skin to a depth of approximately 600 µm, compared with untreated skin (control). Concave indentation of skin surface and disrupted stratum corneum (*arrowhead*). Tips of MN occupied in microchannels, which were dissolved over time (asterisk; *). Scale bars represent 0.5 mm.

Additionally, optical coherence tomography images of the skin after treatment with PRN-loaded MN were obtained. The images are shown in Figure 3. The en-face images and cross-sectional images of the skin, immediately, 4 h and 24 h after MN-application, are shown in Figure 3A,B, respectively. The en-face view shows microchannels formed by PRN-loaded MN until a depth of 600 µm for all time points but most prominent at time points 0 h and 4 h. (Figure 3A).

In the cross-sectional views, the tip of the PRN-loaded MN is even visible in the dermal layer (Figure 3B). PRN-loaded MNs were able to enter the porcine skin reaching a depth of approximately 600 µm in the dermis. After pressing the MN, the stratum corneum was indented and the microchannels were created (Figure 3B; 0 h). Additionally, when comparing the OCT image intensity of adjacent normal dermal tissues, a higher hyperreflectivity of tips of MNs was found within the microchannels (*asterisk*; * in Figure 3B). The microchannels created by MN administration, both in en-face and cross-sectional views at all time points, resemble the arrangement and dimension of the MN array in terms of size of the tip of MN and the center-to-center spacing.

For the closure kinetics of microchannels, the skin indentations at the skin surface were already shallower 4 h after MN insertion. The skin indentations could be resealed almost to their initial condition after 24 h. Nonetheless, a slight discontinuity of the skin surface could still be visualized at the end of the experiment (Figure 3B: 24 h).

#### 3.2.3. Multiphoton Tomography (MPT) with Fluorescence Lifetime Imaging (FLIM)

**Figure 4:**
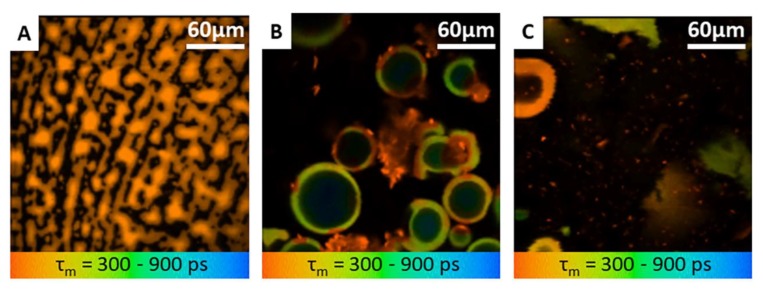
MPT-FLIM images (mean fluorescence lifetime *τ_m_* in the 300–900 ps range) of (**A**) conventional RAL solution (**B**) aggregated PRN suspension (**C**) non-aggregated PRN after sonication (760 nm excitation). Orange coloration indicates the retinal, while yellow-green coloration indicates the nanocarriers; chitosan polymeric nanocarriers. All images acquired at 5 mW at 760 nm. The image size is 124 µm.

Following the insertion of PRN-loaded MN into the skin, MPT-FLIM was utilized to distinguish PRN from dissolved polymer. The PRN signal could be observed primarily in the microchannel of MN treated skin. These false colors are in agreement with the relatively slow autofluorescence lifetime and instant nature of PRN luminescence (Figure 4). 

**Figure 5:**
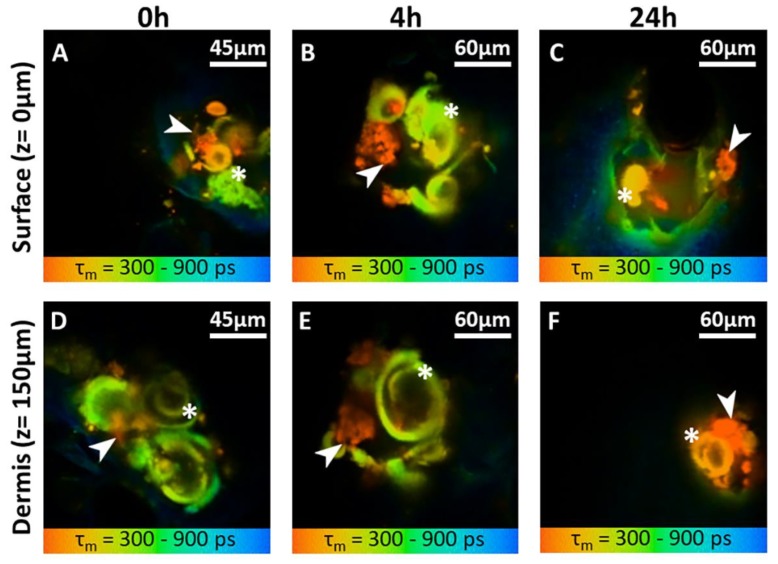
En-face (X-Y scans) MPT-FLIM images (mean fluorescence lifetime *τ_m_* in the 300–900 ps range) of microchannels created by PRN-loaded MN at different time points (0 h, 4 h and 24 h) and at two different depths (skin surface and 150 µm). The deposition of PRN and dissolved polymer from PRN-loaded MN in microchannels (760 nm excitation) at the surface of the skin z = 0 (**A**–**C**) and dermis at the depth of 150 µm (**D**–**F**) at different time points. Orange coloration indicates the aggregation of retinal (*arrowhead*) surrounded by a dissolved polymer and nanocarriers, shown in yellow-green (asterisk; *). All images were acquired at 20 mW and 760 nm.

The microchannels were observed at the skin surface (z = 0 µm) until a depth of 150 µm (z = 150 µm). Some representative MPT images corresponding to one microchannel are shown in Figure 5. The corresponding FLIM images are presented in Figure 5A–F. The main MPT-FLIM features of one microchannel were composed of the well-demarcated and disrupted stratum corneum at the skin surface as the perimeter of the microchannel, dissolved polymer of MN (yellow-green (*asterisk*; *) in Figure 5A–F) and aggregation of PRN (orange (*arrowhead*) in Figure 5A–F). The size of the microchannel openings became slightly smaller within series of time. The openings were approximately 90 µm.

### 3.3. Skin Deposition of Retinal from PRN-Loaded MN, PRN and Conv. RAL

**Figure 6:**
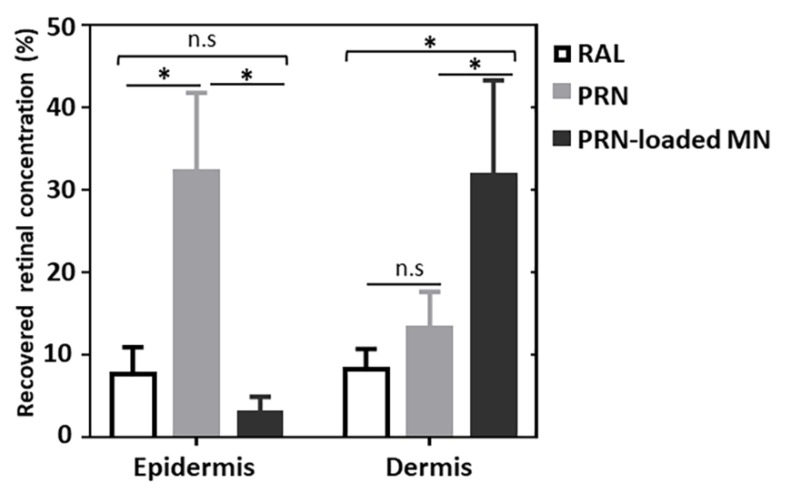
The average percentage of recovered retinal concentrations in epidermis and dermis after topical application as PRN, conventional RAL or PRN-loaded MN. (n = 6, mean ± SD) (* *p* < 0.05 and n.s. for *p* > 0.05).

To compare the concentration of delivered retinal to each skin compartment by different topical formulations (PRN-loaded MN, PRN and conventional RAL), the recovered retinal concentration from epidermis and dermis was quantified as shown in Figure 6. The recovered amount of retinal in epidermis from PRN, conventional RAL and PRN-loaded MN was 0.86, 0.20 and 0.08 µg/cm^2^ or 32.1%, 7.6% and 2.9% from the total amount of the applied formulation, respectively. The amount of retinal recovered from epidermis was highest in the group of topical application of PRN, followed by conventional RAL and lowest from the PRN-loaded MN group. Notably, the recovered concentration of retinal in the epidermis applied by PRN is significantly higher than for the other two formulations (*p* < 0.05) (Figure 6).

The recovered amount of retinal in the dermis from PRN-loaded MN application was 0.85 µg/cm^2^ or 31.7% from the total amount applied, while it was 0.35 µg/cm^2^ or 13.2% for the PRN-treated group and 0.22 µg/cm^2^ or 8.2% for the RAL-treated group. Retinal delivered by PRN-loaded MN was significantly higher compared to the other two groups (*p* < 0.05) (Figure 6).

## 4. Discussion

The stratum corneum, the outermost layer of the skin, consists of densely packed corneocytes, which are embedded in the intercellular lipid matrix, and acts as an efficient barrier against the penetration of topically applied substances. MN are an innovative low-invasive approach to overcome the skin barrier. MN were firstly introduced by Gerstel and Place in 1976 [29]. They suggested that the MN should be long enough to puncture through the stratum corneum and to create a bypass to transport the drug. Until now, the MN technology has been developed in order to facilitate the intradermal delivery of target substances and enhance the skin permeability for nanoparticle [30,31,32].

It has been known for quite a long time that retinoids can have profound effects on the dermis and on the collagen synthesis stimulation. There were some attempts to develop all-trans retinoic acid (ATRA)-loaded microneedles and to confirm the biological activities of ATRA as a safe and effective therapy for seborrheic keratosis and senile lentigo [33,34]. Retinal (RAL) is water insoluble. Attempts to load retinal into the microneedles resulted in needles with liquid oil droplet in the matrix or gel-like needles. Both the MNs with liquid oil droplet and the gel-like needles displayed poor mechanical strength. In a previous study [22], PRN were shown to provide promising physicochemical stabilities, sustain release and less skin irritation than RAL. These findings encouraged us to combine dissolvable tetragonal pyramidal MN and PRN. In a previous study, it has already been shown that PRN is quite stable even when kept as aqueous suspension at 40 °C, shows complete RAL release at 8 h and presents a significantly higher recovery of RAL when compared to non-particulate drug delivery forms [22,23]. Here, the PRNs are embedded in a solid polymeric matrix, sealing them from air oxidation or other degradation transformation. As a result, it was not surprising that we did not observe the color change of the DMNs. This implies the stability of the grafted retinal in the PRN particles which had been embedded in the solid MN matrix, and this shape of MN possesses better mechanical strength than conical shaped MN [35]. It should be noted here that degradation of PRN usually results in color fading.

The results of the present study could confirm that PRN-loaded MN can be inserted through the stratum corneum in ex vivo porcine skin. The disappearance of the tip of the MN embedded in the skin was observed by dermoscopy. It dissolved in the interstitial fluid of the skin due to the water solubility of its casting material, which was polyvinylpyrrolidone and sodium hyaluronate. The dermoscopy was only able to visualize the surface and sub-surface morphological disappearance of PRN-loaded MN and the state of skin.

For investigating the penetration depths of the PRN-loaded MN, OCT images were evaluated. OCT images revealed an MN penetration depth of approximately 600 µm. As the array of PRN-loaded MN was 400 needles/cm^2^ in this study, this was supposed to be enough for effective intradermal drug delivery. Yan et al. investigated the effective needle length and density and demonstrated that microneedles with more than 600 µm in length and a needle density of less than 2000 needles/cm^2^ can efficiently enhance drug flux [36].

After the disruption of the stratum corneum barrier by MN insertion, which results in microchannels, the skin needs to reseal. The microchannels generated by MN are naturally impermanent [37]. In a previous in vivo study, it was suggested that in the absence of occlusion the skin rapidly recovers within 2 h [38]. In the ex vivo skin model of the present study, the microchannels became invisible after 24 h when dermoscopy was applied, but could still be observed by OCT and MPT investigations. Due to the fact that interstitial fluid could be affected by blood perfusion, fluid dynamics or physiological osmotic gradients in the dermis [39], the dissolubility of the casting polymer in the microchannel, especially in an ex vivo model, could be over or underestimated when compared to the living skin. These factors could influence the dissolubility of water-soluble casting polymer. Park and colleagues [40] suggested that their biodegradable polymer microneedles could remain in the skin for several days in order to develop their controlled-release degradation properties and then perform a controlled-release in the skin for months.

MPT provides the possibility to perform label-free imaging and represents an emerging technology in clinical dermatology. MPT permits scanning through the skin from the skin surface down to a depth of 200 μm. In the present study, we successfully demonstrate the existence of PRN in the microchannels after PRN-loaded MN application until 24 h after application. Appearance and FLIM values of PRN in buffer agree with the skin measurements. The fluorescent lifetime is usually reported in the nanosecond range. Fluorescent lifetimes can change with the microenvironment and are optimal to distinguish between PRN and dissolved polymer in microchannels. We hypothesize that PRN, which was released from the MN, can provide a slow release of retinal over time into the dermis, which in turn could reduce the frequency of topical retinal application.

Previously, retinoids have been formulated and topically applied in many different forms [41,42,43,44] including retinal in nanocarrier [22]. In this study, PRN are taken as a model drug for MN-mediated transdermal delivery. Ex vivo retinal deposition in the dermis was significantly enhanced by MN administration. Although PRN have shown to improve the skin conditions of patients [22], theoretically, nanoparticles remain deposited on the outermost layers of the skin [45], in the furrows and wrinkles [46] and in the hair follicles [47,48]. The skin pharmacokinetics of typical retinoids is dependent on a steep concentration gradient [11] as high concentration is achieved in the epidermis, and especially in the stratum corneum, because of its lipophilic properties, resulting in lower concentrations in the dermis. Nanoparticles have been applied in various topical formulations in dermatology. The penetration of different particles is different and also affected by the formulation [49,50]. Up to this point, nanoparticles rarely get into the dermal layer of the skin [51]. In this paper, we use MN as a tool to deliver PRN into the dermis.

Now, it seems possible to include PRN as the drug-loaded polymeric particles into MN tips for direct intradermal drug delivery and targeting exclusively the deeper layers of skin, such as the papillary and even the reticular dermis. The combination of two transdermal drug delivery systems can deliver retinal in nanoparticulate forms to the dermis. As already pointed out, nanoparticles are needed in this application, as it is impossible to fabricate MN loaded with retinal (without being inside the particles) due to the water immersible nature of the drug. The sustained release character of the drug-loaded particles can be of additional benefit. In addition, the ability to incorporate water insoluble drugs into the water-based needle materials using surfactant-free nanoparticles represents a very important feature of the study and various imaging techniques used in this study could help to predict the dissolution rate of RAL from PRN-loaded MN.

PRN could be used as a potential intradermal therapeutic agent to improve atrophic acne scars and to reduce skin aging by stimulating events in the dermis leading to a repair of the damaged skin. They have already been proven to be safe, biocompatible and able to induce a retinoid-based biological activity like epidermal thickening in laboratory rats and human volunteers [22]. Although our proposed system demonstrates a potential benefit and the microneedle application of retinoids into dermis was safe in other studies [33,34], especially the safety of PRN directly applied in the dermis has to be evaluated in further studies.

## 5. Conclusions

This study demonstrated that the combination of MN and PRN, the particulate form of retinal, could enhance the dermal deposition of retinal. These findings could shed light on the possibility of effectively deliver nanoparticles into the dermis reaching maximum therapeutic effects and patient compliance. We hypothesize that this platform of PRN-loaded MN can provide rapid drug administration to the dermis after MN insertion and could thus be beneficial in some skin conditions such as atrophic scar and photo aged skin in the future.

## Supplementary Materials

The following are available online at https://www.mdpi.com/2079-4991/10/2/368/s1, Figure S1: SEM image reveals spherical PRN particles with the dry size of around 1 µm, Figure S2: En-face MPT images (autofluorescence mode, X-Y scans) of microchannels created by PRN-loaded MN at different time points and at two different depths.
