# Supplementary figures and images for: Microneedle-Facilitated Intradermal Proretinal Nanoparticle Delivery
Source: Nanomaterials (Basel). 2020 Feb 20;10(2):368. doi: 10.3390/nano10020368 (PMC7075281; doi:10.3390/nano10020368)

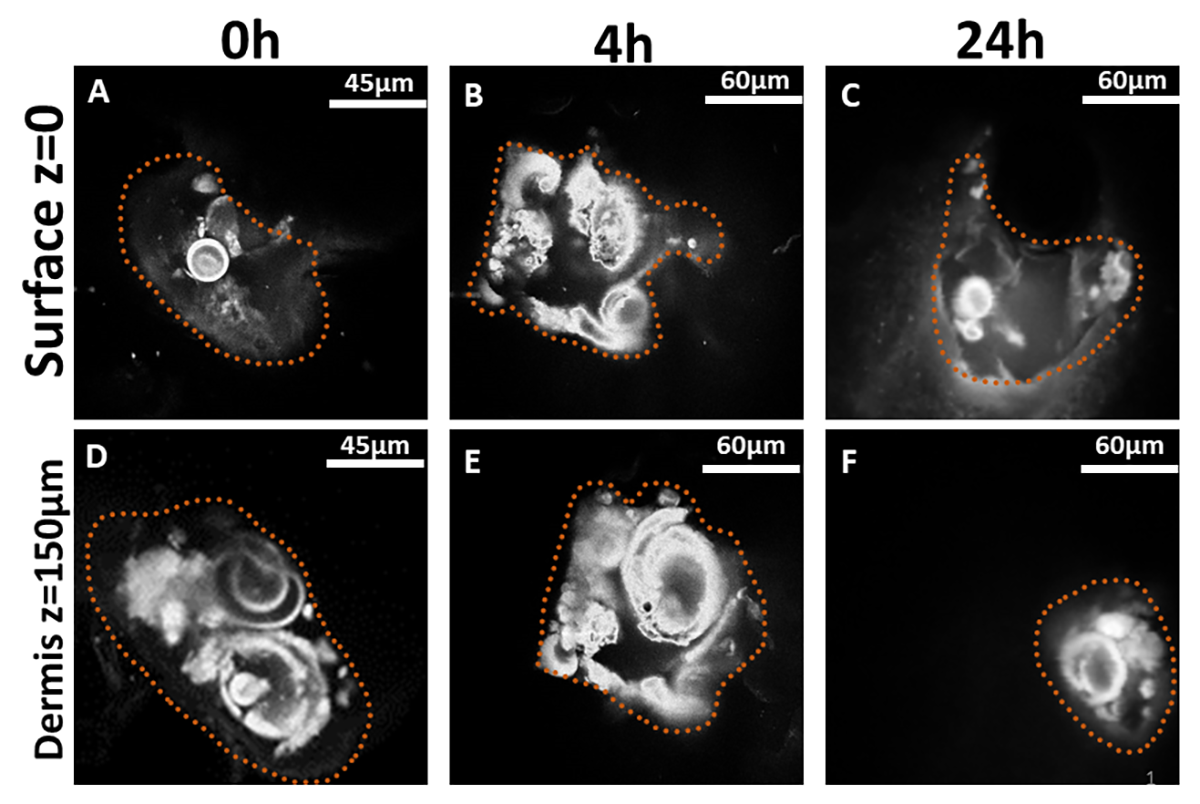

Supplement: Supplementary file 1 [file nanomaterials-10-00368-s001.zip › FigS_Autoflu_MN.tif]

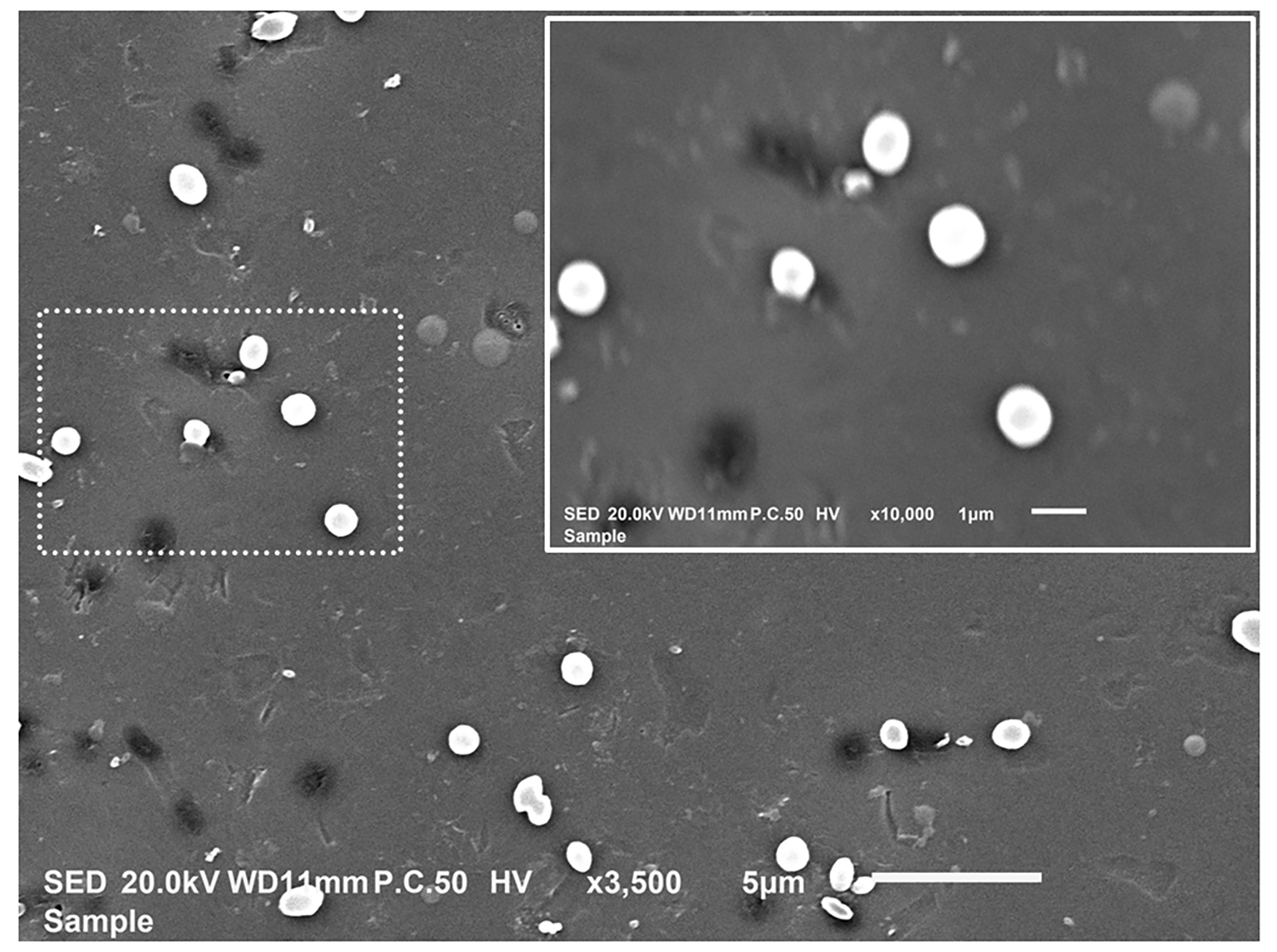

Supplement: Supplementary file 1 [file nanomaterials-10-00368-s001.zip › FigS_SEM_dryspherePRN.tif]
